# Intratumoral heterogeneity of *c-KIT* mutations in a feline splenic mast cell tumor and their functional effects on cell proliferation

**DOI:** 10.1038/s41598-022-19089-5

**Published:** 2022-09-22

**Authors:** Yuki Hasegawa, Kazuha Shosu, kanako Tsuji, Yumiko Shimoyama, Takako Shimokawa Miyama, Kenji Baba, Masaru Okuda, Kazuhito Itamoto, Masaya Igase, Takuya Mizuno

**Affiliations:** 1 Laboratory of Molecular Diagnostics and Therapeutics, Joint Faculty of Veterinary Medicine, Yamaguchi University, Yamaguchi, Japan; 2 Laboratory of Veterinary Internal Medicine, Joint Faculty of Veterinary Medicine, Yamaguchi University, Yamaguchi, Japan; 3 IDEXX Laboratories, Tokyo, Japan; 4 Laboratory of Small Animal Clinical Science, Joint Faculty of Veterinary Medicine, Yamaguchi University, Yamaguchi, Japan

**Keywords:** Cancer therapy, Cancer, Molecular biology

## Abstract

A cat was presented with mast cell tumors (MCTs) of the skin and spleen. During the initial diagnosis, the exon 8 mutation of *c-KIT* was detected in the masses from skin and spleen by a commercial laboratory test. Consequently, treatment with toceranib was started. After complete remission, because of recurrence on day 117, the spleen and skin tumors were removed, but the cat eventually died on day 191. The analysis of ten cDNA clones of the *c-KIT* gene cloned from the surgically removed spleen revealed that seven different cDNA patterns were included, indicating the heterogeneity of this gene in the splenic MCT. The seven cDNA nucleotide patterns can be classified into four protein sequence patterns. In addition to the previously known mutations in exon 8, we identified novel mutations in exons 9, 10, and 18; four amino acids deletion in exon 9, and a point mutation in exons 10 and 18. Mouse IL-3-dependent cell line, Ba/F3, was transduced with these mutant clones, and c-KIT phosphorylation and proliferation assays were performed. We found that certain mutations affected the c-KIT phosphorylation status and cell proliferation. This suggests that heterogeneity among the population of tumor cells exists in MCTs, and that the dominant clones of this heterogeneity may contribute to the subsequent tumor cell growth.

## Introduction

Feline mast cell tumor (MCT), which is the most common tumor of the spleen, is a frequently encountered tumor in cats, especially in the skin^1^. Mutations in the *c-KIT* gene, receptor tyrosine kinase expressed on mast cells, have been reported in tumor cells of feline MCT^2–5^, and this is believed to be one of the causes of feline MCT. A variety of *c-KIT* mutation patterns have been reported; however, information on *c-KIT* mutations in feline MCTs remains little. Since insertional mutations in exon 8 are the most frequently observed^4,6^, only a limited number of exons, mainly exons 8 and 9, have been investigated. Furthermore, whether individual mutations that have been previously reported are truly linked to c-KIT activation or tumor cell proliferation is unclear.

Mutations in the *c-KIT* gene are found in mastocytosis and gastrointestinal stromal tumors (GISTs) in humans and in 20–30% of canine MCT^6^. Activating mutations in the *c-KIT* leads to MAPK activation^7^, which in turn causes tumor cell growth. Consequently, molecularly targeted drugs inhibiting c-KIT activation have been successfully used as therapeutic agents not only in GIST in humans^8^ but also feline MCT^6^. Therefore, identifying key information about *c-KIT* mutations and evaluating their role in the subsequent pathogenesis are very important. Several reports showed that the mutations of feline *c-KIT* ^9^, and exons 8, 11, and 12 of feline *c-KIT* are often tested with commercial laboratory tests, whereas mutations in other sites are generally not examined because their contributions are perceived as less significant. Hence, a broad analysis of *c-KIT* mutations in feline MCT is very important for obtaining clinical information. In addition, it is also known that intratumor heterogeneity may be associated with tumor cell growth and selection of some tumor cells by treatment^10^.

In this study, we isolated and cloned 10 cDNA clones of feline *c-KIT* from a cat with splenic MCT. By analyzing the nucleotide sequences of these clones, we identified the existence of multiple *c-KIT* clones, some of which were not previously reported in literature. Among them, certain mutations were missense mutations, i.e., causing the substitution of a single amino acid. To analyze the function of mutant *c-KIT* genes, the mutant proteins were exogenously expressed in mouse IL-3-dependent Ba/F3 cell lines, and some of them were proved to have functions of induction of c-KIT activation and cell proliferation.

## Results

### Case description

A 16-year-old spayed female sham cat was presented for a detailed examination of several skin masses. Physical examination revealed that six masses were present in the entire body skin, including one located at the root of the left ear, whereas the remaining masses were located on the trunk. The mass on the root of the left ear appeared one year ago, but other masses appeared just two months before initial administration to our hospital. Blood examination showed no abnormalities. Splenomegaly was identified by abdominal ultrasonography. The fine needle aspiration of the spleen allowed us to diagnose this cat with MCT in the spleen. One of the skin masses was punch-biopsied on the same day, and it turned out to be a high-grade MCT. Using the samples from skin biopsy and spleen, the exon 8 insertion (c.1245-1256dup) of *c-KIT* was detected by a commercial laboratory test without any other mutations in exons 9 and 11.

According to the above diagnosis, we started the administration of toceranib (2.3 mg/kg) every other day and diphenhydramine (2.3 mg/kg) B.I.D. At recheck on day 19, all masses on the skin completely disappeared, followed by the disappearance of splenomegaly. However, on day 105, the mass on the root of the left ear grew again, and this mass was removed by surgery on day 117. Splenectomy was also performed on the same day. The histopathological findings of the ear mass and spleen revealed a high- and low-grade MCT, respectively, and the *c-KIT* mutation analysis by commercial laboratory demonstrated that both tumors had the same *c-KIT* mutation (exon 8 insertion), as shown in the initial presentation. Following the removal of the spleen and skin tumors, the cat was under observation at the local doctor, and eventually died on day 191.

### cDNA analysis of c-kit mutation in the spleen

**Figure 1:**
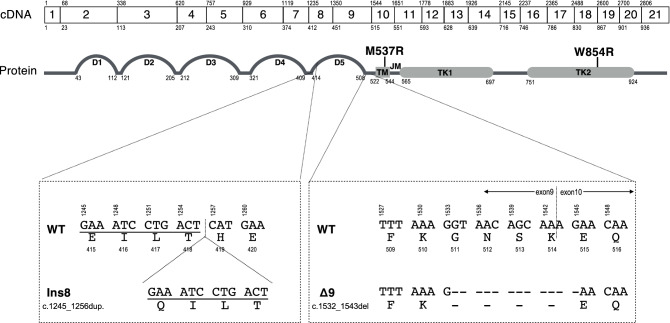
Schematic representation of the structure of feline *c-KIT* gene and c-KIT protein. Boxes show the exon structure of feline *c-KIT* cDNA, and the numbers in each box indicate the exon number. The number above and below each box indicates the numbers of the first nucleotide and amino acid in each exon, respectively. Protein structure is shown below the DNA structure in gray line and gray boxes, as predicted from the amino acid sequence of feline c-KIT by InterPro (https://www.ebi.ac.uk/interpro/). Numbers below the protein structure indicate the amino acid numbers of each domain. D1–D5 refer to immunoglobulin domains. TM, transmembrane domain; JM, juxtamembrane domain; TK, tyrosine kinase domain. M537R and W854R indicate the point mutation in the TM domain and TK2 domain, respectively. Dotted boxes below the protein structure represent the mutations in exons 8 and 9. WT, wild type; Ins8, the insertional mutation in exon 8; Δ9, the deleted mutation in exon 9.

Next, 10 independent *c-KIT* cDNA clones were obtained from a spleen cDNA sample and were sequenced. As shown in Table 1, seven different clones (cDNA pattern A–G in Table 1) were identified in a spleen sample, including four cDNA type A clones, and each of cDNA pattern B, C, D, E, F, and G clones. Comparison with the registered sequence of feline *c-KIT* cDNA (NM_001009837.3), all these clones had the same silent mutation (c.2856G > A) in exon 21 and deletion of 12 nucleotides (c.1532_1543del) in exon 9 (Fig. 1), leading to a lack of 4 amino acids (GNSK), but no frameshift. Other several silent mutations were also found in exons 3 and/or 10 in several clones (Table 1). Furthermore, the duplication of 1245–1256 in exon 8 (c.1245_1256dup.) was found in cDNA patterns A, B, C, D, and E. We also found the unreported point mutations in exons 10 and 18, in cDNA types E and G, respectively, both of them being missense mutations (Fig. 1). Consequently, this analysis facilitated a classification into four types of aberrant c-kit proteins, i.e., patterns I to IV, of which type I was predominant in clones, and types II, III, and IV indicated the minor clones (Table 1).

**Table 1:** Summary of the mutations found in cDNA analysis from the spleen of patient cat.

cDNA pattern	Numbers of each clone	Exon3		Exon8	Exon9	Exon10			Exon18	Exon21	Protein pattern
		c.396A > G	c.531T > C	**c.1245_1256dup (Ins8)**	**c.1532_1543del (Δ9)**	c.1579C > T	**c.1610T > G (M537R)**	c.1617T > C	**c.2560T > C (W854R)**	c.2856G > A	
A	4			** + **	** + **					+	I (Ins8-Δ9)
B	1	+	+	** + **	** + **					+	
C	1			** + **	** + **			+		+	
D	1	+	+	** + **	** + **			+		+	
E	1	+	+	** + **	** + **		** + **			+	II (Ins8-Δ9-M537R)
F	1	+	+		** + **			+		+	III (Δ9)
G	1	+	+		** + **	+			** + **	+	IV (Δ9-W854R)

+ (plus) indicated the mutations found in cDNA clones, whereas empty columns indicated no mutation.

Bold values indicated the mutations resulting in changes of amino acids.

### Effect of *c-KIT* mutations on cell proliferation

**Figure 2:**
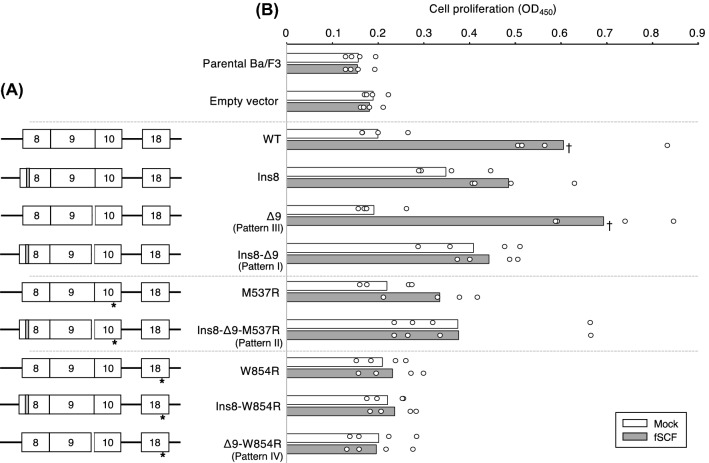
Cell proliferation rate of Ba/F3 cell lines expressing mutant c-KIT proteins. (**A**) Schematic representation of mutation patterns of *c-KIT* clones used for this assay. Asterisk indicates the point mutation in exon 10 (M537R) or the point mutation in exons 18 (W854R). The gray box and the blank between exons 9 and 10 indicate the duplicated mutation in exon 8 and the four amino acids deletion in exon 9, respectively. (**B**) Cell proliferation rate of Ba/F3 cell lines expressing each mutant c-KIT protein. Each mutant cell line was cultured for 48 h in the medium, without (mock, white bar) or with feline SCF (fSCF; gray bar). After 48 h, CCK-8 was added, and the cell proliferation rate was measured by plate reader. Bar graph indicates the mean from four independent biological replicates, and open dots indicate the values obtained from each experiment. *p*-values were calculated for the comparison among all cell lines using two-tailed one-way ANOVA with a post hoc Tukey–Kramer test. Dagger indicates a significant difference (*p* < 0.0001) between Mock and fSCF-treated each cell line.

Among these identified aberrant *c-KIT* cDNAs, c.1245_1256dup. in exon 8 was already known as a *c-*KIT active mutation^2^. However, the functional effects of the other mutations found in this study have not yet been reported. To elucidate the functional impact of these mutations, several stable transfectants of mouse IL-3-dependent Ba/F3 cell lines expressing either of these aberrant c-KIT proteins were established. Figure 2A shows the schematic lists of aberrant c-KIT proteins, I to IV cloned from this patient cat (patterns I–IV). In addition, we have also generated a wild-type feline *c-KIT* expressing plasmid (WT), and the four other kinds of plasmids having one of the Ins8, M537R, W854R, or Ins8-W854R mutations, which would help to identify the true functions of M537R and W854R mutations.

Using the aberrant *c-KIT*-transduced Ba/F3 stable cell lines, we examined the effect of these mutations on cell proliferation (Fig. 2B). Since the Ba/F3 cell line grows only in the presence of mouse IL-3, we examined the effect of aberrant c-KIT proteins on the cell proliferation of Ba/F3 cell lines expressing one of aberrant c-KIT proteins with or without feline SCF (fSCF). Ba/F3/WT cells did not proliferate without mouse IL-3 but did proliferate in the presence of fSCF. As Ins8 mutation proved to be active mutation of *c-KIT*, even in the absence of fSCF, Ba/F3/Ins8 tended to proliferate more than Ba/F3/WT, but not significant. Δ9 mutation was never reported before in dogs and cats, but Ba/F3/Δ9 also exhibited a similar proliferative pattern as Ba/F3/WT, and this Δ9 mutation did not appear to be affecting the growth. Ba/F3/Ins8-Δ9 without fSCF also tended to proliferate higher than Ba/F3/WT and Ba/F3/Δ9, as similar as Ba/F3/Ins8. This result indicated that the Ins8 mutation seemed to have growth advantage. M537R mutation showed no effect on cell proliferation as shown in Ba/F3/M537R and Ba/F3/Ins8-Δ9-M537R.

Finally, we validated the surprising effect of W854R mutation on cell proliferation. Irrespective of the presence of fSCF, cell proliferation was completely abrogated in the Ba/F3/W854R, Ba/F3/Ins8-w854R and Ba/F3/Δ9-W854R cell lines, as opposed to the Ba/F3/WT, Ba/F3/Ins8 and Ba/F3/Δ9 cell lines. This indicated that the W854R mutation completely suppressed the c-KIT-dependent cell proliferation.

### Effect of *c-KIT* mutations on c-KIT phosphorylation

**Figure 3:**
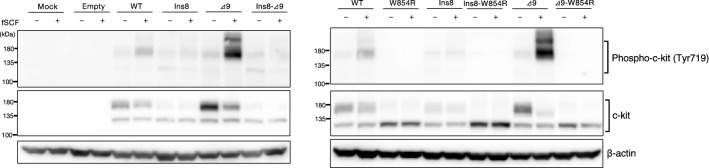
Phosphorylation status of c-KIT in Ba/F3 cells expressing the mutant *c-KIT*. Ba/F3 cell lines stably expressing mutant forms of c-KIT protein were cultured in plain RPMI for 2 h and stimulated with fSCF (100 ng/ml) for 5 min. After stimulation, cells were collected and the extracted whole cell lysates were conducted for SDS-PAGE, followed by western blotting with anti-phospho-c-KIT (Tyr719), anti-Flag (M2), and anti-β-actin antibodies. A representative result of three independent experiments is shown. Full image of western blotting is shown in Supplementary Figure.

Next, we examined the effect of these mutations on the c-KIT activation status. For that purpose, phosphorylated c-KIT at Tyr719 was detected by western blotting in each Ba/F3 cell line before and 5 min after fSCF stimulation. As shown in Fig. 3, in Ba/F3/WT cells, the phosphorylation of c-KIT (mature type, 140 kDa) was induced by stimulation with fSCF. Ba/F3/Ins8 showed almost similar phosphorylation of c-KIT mature type, but we also observed the immature form of phosphorylated c-KIT even in the absence of fSCF. The addition of fSCF in Ba/F3/Δ9 induced the phosphorylation of mature c-KIT, but much more profound than Ba/F3/WT and Ba/F3/Ins8. Moreover, Ba/F3/Ins8-Δ9 showed the quite similar pattern of Ba/F3/Ins8, and the profound phosphorylation of mature c-KIT shown in Ba/F3/Δ9 has not been observed anymore.

We also examined the effect of W854R mutation on the c-KIT phosphorylation. Surprisingly, all of the phosphorylated c-KIT completely disappeared irrespective of the timing (before or after) of fSCF stimulation.

### Effect of tyrosine kinase inhibitor on cell proliferation of *c-KIT*-mutated Ba/F3 cells

**Figure 4:**
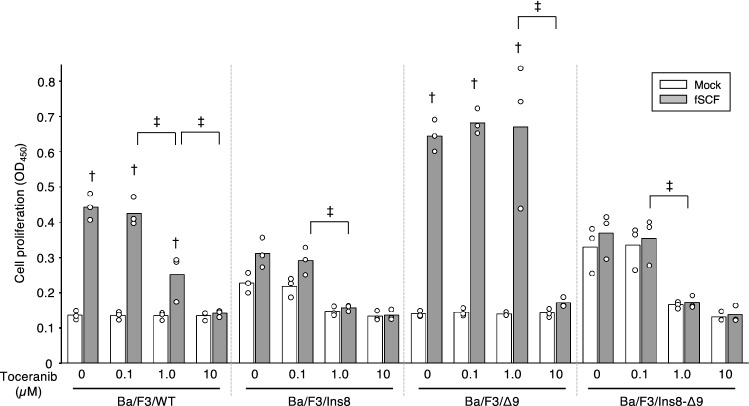
The effects of toceranib on cell proliferation rate of Ba/F3 cell lines expressing mutant c-KIT proteins. Cell proliferation rate of Ba/F3 cell lines expressing each mutant c-KIT protein was examined with the different amounts of toceranib (0, 0.1, 1.0 and 10 µM). Each mutant cell line was cultured for 48 h in the medium (mock, white bar) or with fSCF (gray bar). After 48 h, CCK-8 was added, and the cell proliferation rate was measured by plate reader. Bar graph indicates the mean from three independent biological replicates, and open dots indicate the values obtained from each experiment. *p*-values were calculated for the comparison among all cell lines using two-tailed one-way ANOVA with a post hoc Tukey–Kramer test. Dagger indicates a significant difference (*p* < 0.05) between with or without fSCF, within each cell line. Double dagger indicates the significance (*p* < 0.05) between the different dosages within fSCF-treated each cell line.

We also assessed the effect of the toceranib on cell proliferation of these Ba/F3 mutant cell lines (Fig. 4). Feline SCF-induced Ba/F3/WT proliferation was dose dependently inhibited by toceranib treatment, and 1 µM was not sufficient to block the proliferation completely. However, Ba/F3/Ins8 cells proliferation was completely inhibited by toceranib treatment even at 1 µM. Furthermore, Ba/F3/Δ9 cells were not influenced at all by the treatment of 0.1 and 1 µM of toceranib, and needed 10 µM of toceranib to have a suppressed cell proliferation. Surprisingly, Ba/F3/Ins8-Δ9 reflected the similar trends of effects of toceranib on cell proliferation, as consistent with the result seen in Ba/F3/Ins8.

## Discussion

In this report, we identified the novel mutations of the *c-KIT* gene from the splenic tumor sample of a feline MCT, in addition to the common exon 8 insertional mutations. In addition, this cat had skin masses that were diagnosed as MCT with the same exon 8 mutations by a commercial laboratory test. We started the subsequent treatment with toceranib, and MCT in all of the skin sites disappeared. Furthermore, ultrasonography revealed an improvement in the splenomegaly and echogenicity of the spleen. However, the recurrence of skin MCT appeared after 100 days, and the cat died on day 191. This is consistent with the findings of Sabattini et al*.* reported that cats with skin and spleen MCT tended to die earlier than cases with an exclusive splenic involvement^4^. Exon 8 insertional mutations were detected by a commercial laboratory test in the samples both before and after the treatment, including skin mass and spleen, although other mutations, such as S477I and N506I in exon 9, tested by a commercial laboratory, were not observed. However, the range of commercial laboratory test was practically limited to duplicate mutations in exon 8 and these point mutations in exon 9, suggesting that any other mutations of *c-KIT* were unknown.

Intratumor heterogeneity is one of the characteristic features of tumors that is often related to treatment resistance^11^, especially in molecularly targeted therapy, and reflects the heterogeneous proliferation. In this study, we identified several different clones from spleen MCT. We isolated five types of independent cDNA clones (one major clone and four minor clones) from ten clones. These clones were isolated from only a small part of splenic tissues. However, had we used the different parts of splenic tissues, we could have had more types of clones. Furthermore, we obtained splenic tissue for sequence analysis after the beginning of the toceranib therapy. Therefore, we cannot exclude the possibility that molecularly targeted therapy facilitated this type of heterogeneity during treatment, which may have, in turn, led one major clone to obtain a cell growth advantage or exhibit resistance to the therapy. In dogs and cats, *c-KIT* mutations are often reported, but to date, there are no reports on intratumoral heterogeneity. The intratumoral heterogeneity of *c-KIT* mutation was reported in human GIST^12^, and that of p53 mutation was reported in canine histiocytic sarcoma^13^. Recently, next-generation sequence technology and single-cell analysis methods are becoming increasingly popular. Consequently, these technologies can provide researchers with more precise information regarding heterogeneity studies.

In this study, the cat had several skin MCTs in addition to splenic MCT. Although commercial laboratory tests showed the same mutation of exon 8, we could not compare the sequence similarity of other *c-KIT* mutations between spleen and skin tumors, even among several skin tumors, because tissue samples for RNA extraction were unavailable. The *c-KIT* sequences of genomic DNA were also not examined by ourselves, because they were also unavailable. The comparison of genomic DNA in tumor and normal samples would provide us with the information of whether the mutations found in our study were generated intratumorally generated, skin tumor or spleen tumor that metastasized to one another, or whether they came as stemmed from intratumoral heterogeneity as reported in multiple skin MCTs in dogs^14^.

We found several new mutations that were not previously reported. c.1532_1543del mutation in exon 9 was found in all clones, indicating that this mutation may be a genomic mutation or somatic mutation obtained by MCT at the early stages of tumorigenesis. c.1532_1543del mutation in exon 9 generated the deletion of amino acids GNSK, which corresponded to the GNNK-deletion in the juxtamembrane region of the extracellular domain, often found in human and mouse c-KIT as an alternative splicing isoform. GNNK deletion is known to be induced stronger and rapid phosphorylation of c-KIT^15^, as consistent with predominant strong phosphorylation of c-KIT in Ba/F3 cell lines having Δ9 mutations in our study. However, this strong phosphorylation did not induce additive proliferation effect as compared with Ba/F3 wild type cells, but this mutation showed relative resistance to toceranib treatment at 1 µM, though Ba/F3/WT, Ba/F3/Ins8, and Ba/F3/Ins8/Δ9 had sensitivity to toceranib at 1 µM to some extent. This indicated that Δ9 mutation had some functional effect on c-KIT activation, but need to be clarified in the future.

c. 2560T > C mutation was found in only one clone, and this is quite a unique mutation. This led to W854R mutation, which suppressed the c-KIT activation and cell proliferation induced by c-KIT activation. In canine and feline MCT, there have been no reports on the suppressive mutation of c-KIT activation, perhaps because researchers have not attempted to examine the mutations in exon 18 using c-kit analysis. In human MCT, the E839K type of suppressive mutation was previously reported^16^. E839K is located in the tyrosine kinase domain, which is quite similar to the c-kit protein of the W854R mutation. As shown in Figs. 2 and 3, this mutation completely suppressed the c-kit activation induced by exon 8 insertion mutation. Generally, this mutation does not provide a growth advantage to the tumor; thus, a clone having this mutation would be a minor clone in the tumor. This in turn suggests that this mutation has a smaller clinical impact.

We also found an M537R mutation in exon 10, where was located in the transmembrane region of the c-KIT by hydrophobicity analysis. Few *c-KIT* mutations in the transmembrane domain have been identified, and F522C mutation in the transmembrane domain of the human c-KIT was reported in a patient with mastocytosis^17^. F522C mutation caused the autophosphorylation of c-KIT, which is sensitive to the treatment with imatinib; this means that this mutation is responsible for cell proliferation. In this study, M537R mutation showed no advantage of cell proliferation in the absence of any cytokine, despite the fact that it seemed to influence the increase of immature form of c-KIT without fSCF. But true function of this mutation remained unclear.

In this study, the relationship of the phosphorylation at Tyr 719 of c-KIT and c-KIT-dependent cell proliferation has not been clarified, although the functional effects of each mutant of feline *c-KIT* on cell proliferation was revealed, which was first report about feline *c-KIT*. The phosphorylation at Tyr 719 of c-KIT may not represent the true c-KIT activation related to the cell proliferation effect. Instead, analyzing the other phosphorylated sites or c-KIT activation status may be a good indicator of c-KIT activation showing the phenotypic function such as cell proliferation.

In conclusion, we identified new *c-KIT* mutations in feline MCT and analyzed their functions. In addition, the presence of heterogeneity in feline MCT was revealed. It is expected that more comprehensive analysis of the *c-KIT* gene will be conducted in the future to clarify the pathogenesis of MCT in cats.

## Methods

### Cell line validation statement and culture

PLAT-gp cell line was kindly provided by Dr. Toshio Kitamura (Institute of Medical Science, University of Tokyo, Japan) and was grown in D10 complete medium (Dulbecco's modified eagle medium (DMEM) supplemented with 4500 mg/L high glucose, 10% fetal bovine serum, 100 U/ml penicillin, 100 μg/ml streptomycin, and 55 μM 2-mercaptoethanol)^18^. Ba/F3 cell line, which was obtained from the Cell Resource Center for Biomedical Research (Institute of Development, Aging and Cancer, Tohoku University, Sendai, Japan), was grown in R10 complete medium (RPMI1640 supplemented with the same reagents as D10 complete medium) plus 10 ng/ml of mouse IL-3 (PeproTech, Cranbury, NJ, USA). All cell lines were maintained at 37 °C in a humidified 5% CO_2_ incubator.

### Molecular cloning of feline c-kit cDNAs

After obtaining written consent from the owner for the use of the residual sample of the spleen, which is the surgical specimen for treatment, total RNA was isolated from the spleen sample using the ISOGEN II kit (NIPPON GENE, Tokyo, Japan), and cDNA was synthesized using the Superscript III first-strand synthesis system for RT-PCR (Thermo Fisher, Tokyo, Japan), according to the manufacturer's instructions. Feline *c-KIT* cDNA was amplified with primers, YTM1483 (5′-CAGGAACGTGGAACGGACCTC-3′^2^) and YTM1509 (5′-TCTACCCTGGAACAGGATGC-3′) by PCR. The resultant PCR products were electrophoresed in agarose gel, and the expected band was extracted, followed by second PCR with YTM1527 (5′-ACGGATCCCAGGAACGTGGAACGGACCTC-3′, BamHI site was underlined)-YTM1528 (5′-AACTCGAGGATCGTTCTCGCTGGGGAGAC-3′, XhoI site was underlined) to carry BamHI-XhoI sites on both ends. The products were digested with BamHI and XhoI and cloned into BamHI-XhoI sites of pcDNA3 plasmid vector. The nucleotide sequence analysis of each plasmid was performed by the DNA Core Facility of the Center for Gene Research, Yamaguchi University, and compared with the nucleotide sequence of feline c-kit registered in the public database (NM_001009837.3).

### Construction of mutant c-kit vectors and retroviral expression vectors

To obtain retroviral expression vectors, all cDNA clones in pcDNA3 were transferred into the pMx-IP vector (kindly provided by Dr. Toshio Kitamura) with FLAG-tag sequences at C-terminus. Furthermore, to obtain the other mutant forms of feline c-kit, which were not cloned in this study, PCR-based molecular techniques were used to transduce the desired mutations. All mutations were confirmed by nucleotide sequence analysis.

### Establishment of Ba/F3 cell lines overexpressing mutant feline *c-KIT*

Ba/F3 cell line was retrovirally transduced with mutant feline *c-KIT* vectors. Briefly, PLAT-gp cell lines were transfected with one of the *c-KIT* expression vectors and pCAGGS-VSVG using PEI max. After 48 h, the supernatant was collected and added to Ba/F3 cell line for infection. Finally, *c-KIT* -transduced Ba/F3 cell lines were selected by 4 µg/ml of puromycin.

### Cell proliferation assay

For the cell proliferation assay, each Ba/F3 cell line was seeded in triplicate in 96-well plates at 5.0 × 10^3^ cells/well, in the presence of 10 ng/ml of mouse IL-3 or 100 ng/ml feline SCF (fSCF; R&D Systems, Inc., Minneapolis, MN), and cultured for 48 h. After 48 h of incubation, CCK8 reagent (DOJINDO, Kumamoto, Japan) was added to each well, and cells were cultured for an additional 1 h. Following this 1 h incubation, the absorbance at 450 nm was measured in the plates using the ARVO (Perkin Elmer) plate reader. The same experiments were repeated three times.

### Western blotting

To evaluate the activation status of the mutant *c-KIT*, each cell line was seeded in 6-well plates at 5.0 × 10^5^ cells/well and incubated for 3 h in the plain RPMI. After incubation, cells were stimulated with fSCF (100 ng/ml) for 5 min and harvested and lysed with NP40 lysis buffer [1% NP 40, 10 mM Tris HCL (pH 7.5), 150 mM NaCl, 1 mM EDTA, protease inhibitor cocktails (Nacalai Tesque, Kyoto, Japan), 1 mM Na_3_VO_4_, and 50 mM NaF]. The resulting protein lysates were loaded on SDS–polyacrylamide gels and electrophoresed followed by blotting to polyvinylidene fluoride transfer membrane (Merck, Darmstadt, Germany). Then, the membrane was blocked with 5% skim milk blocking buffer, followed by incubation with the primary antibody, rabbit polyclonal anti-phospho-c-KIT antibody (Tyr719) (Cell Signaling Technology, Danvers, MA, USA), goat polyclonal anti-c-KIT antibody (M-14, Santa Cruz Biotechnology), or mouse monoclonal anti-beta actin antibody (Sigma-Aldrich). Horseradish peroxidase (HRP)-conjugated antibodies, including donkey anti-rabbit IgG-HRP (Jackson ImmunoResearch Laboratories, West Grove, PA, USA), goat anti-mouse IgG-HRP (Bio-Rad Laboratories) and goat anti-mouse IgG-HRP (Bio-Rad Laboratories) were used as secondary antibodies. The specific proteins were detected using the ECL reagent (PerkinElmer, Waltham, MA, USA).

### Statistical analysis

Each assay was performed as three technical replicates and repeated as a series of at least three independent biological replicates. All of the calculated data were compared using a two-tailed one-way factorial ANOVA test followed by multiple comparisons using the Tukey–Kramer HSD test (for comparisons of more than two groups). Calculations were performed using the JMP software, ver. 14.0 (SAS Institute Japan, Tokyo, Japan). A *p-value* of less than 0.05 was considered significant.

## Supplementary Information

Supplementary Information.

## Data Availability

The data that support the findings of this study are available from the corresponding author upon reasonable request.
