# Supplementary figures and images for: Intratumoral heterogeneity of c-KIT mutations in a feline splenic mast cell tumor and their functional effects on cell proliferation
Source: Sci Rep. 2022 Sep 22;12:15791. doi: 10.1038/s41598-022-19089-5 (PMC9499958; doi:10.1038/s41598-022-19089-5)

# Supplementary figures for Fig. 3

Fig. 3

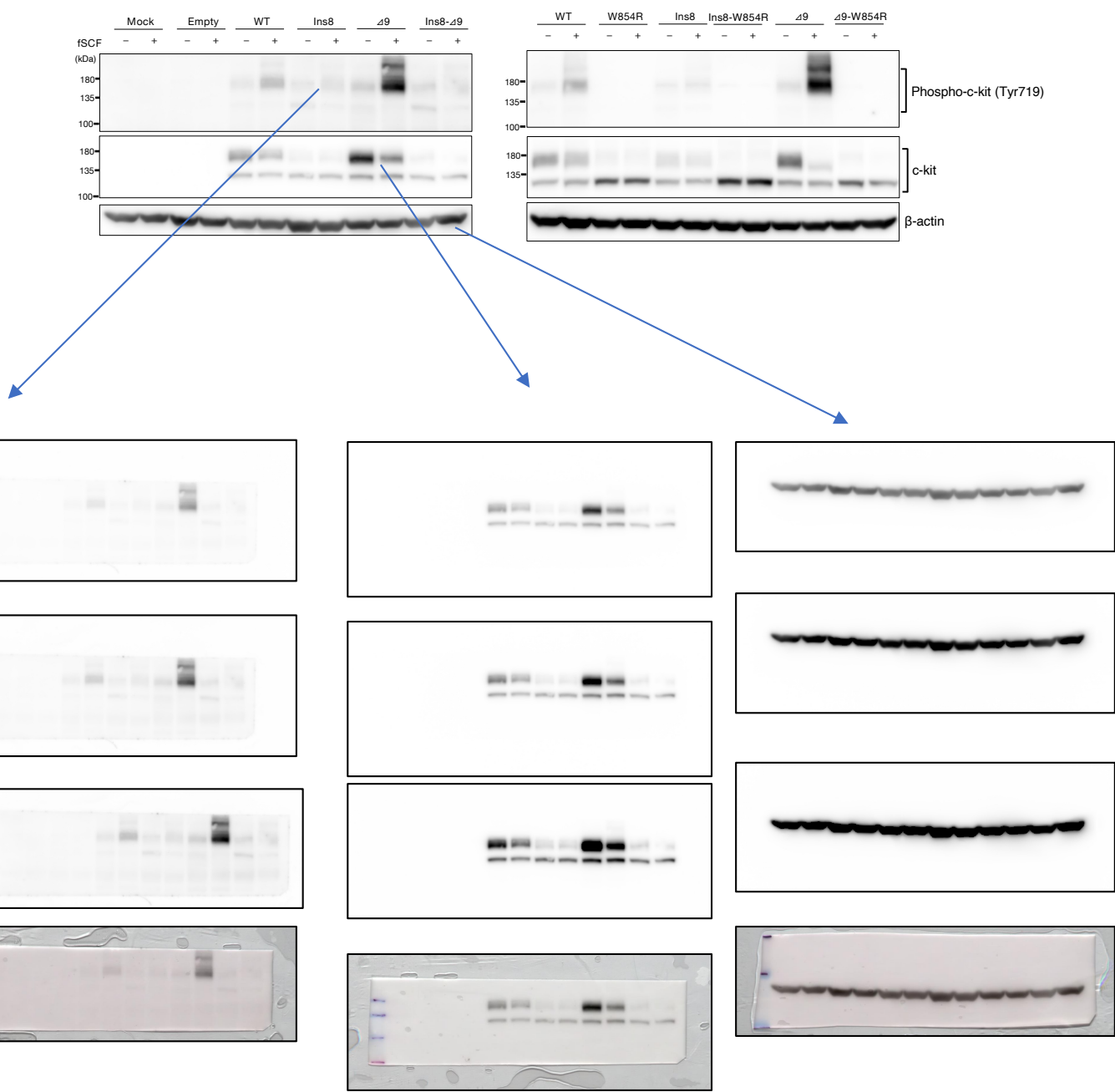

Supplementary figures for Fig. 3

Fig. 3

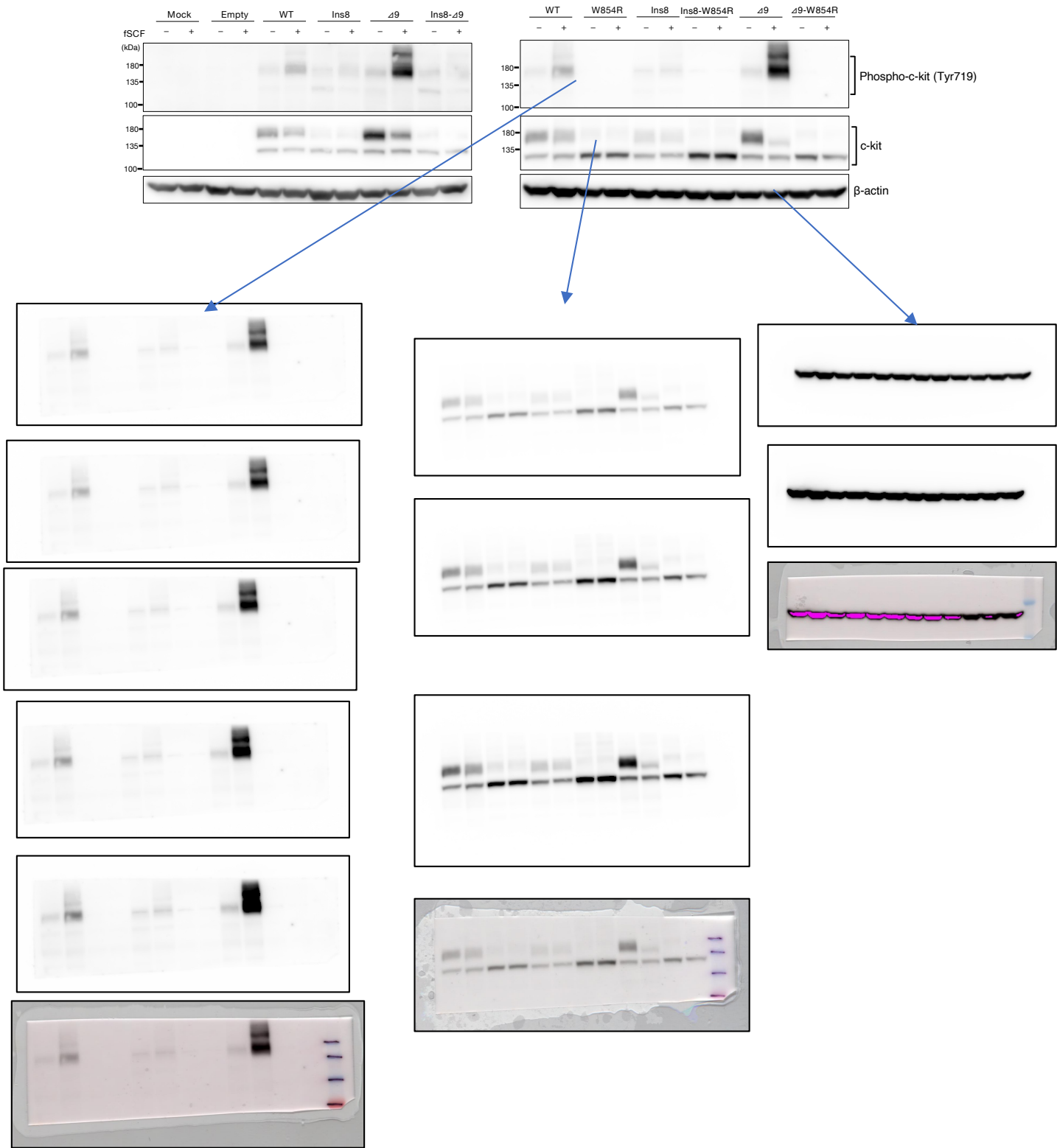

Supplement: Supplementary file 1 — Supplementary Information. [file 41598_2022_19089_MOESM1_ESM.pdf]
